# Skin diseases in hospitalized geriatrics: a 9-year analysis from a University Dermatology Center in Germany

**DOI:** 10.1007/s00403-021-02244-9

**Published:** 2021-06-02

**Authors:** Claudia Ansorge, Johannes M. Miocic, Franziska Schauer

**Affiliations:** 1 Department of Dermatology and Venereology, Medical Center, Faculty of Medicine, University of Freiburg, Hauptstraße 7, 79104 Freiburg, Germany; 2 Institute of Earth and Environmental Sciences, University of Freiburg, Freiburg, Germany; 3 Geo-Energy, Energy and Sustainability Research Institute Groningen, University of Groningen, Nijenborgh 6, 9747 AG, Groningen, The Netherlands

**Keywords:** Dermatology, Epidemiology, Geriatric patients, Inpatients, Comorbidities, NMSC

## Abstract

The demographic trend of an ageing society is mirrored in the rising number of hospitalized geriatric patients in Germany. However, there is still a wide gap of knowledge regarding the dermatological diseases, comorbidities and performed procedures within this growingly important group of patients. The study was conducted as a retrospective monocentric data analysis of all patients 65 years or older from the Department of Dermatology, Medical Center—University of Freiburg, Germany. In total, 10,009 individual hospitalisations were included from 2009 to 2017, and there was a notable increase of geriatric patients in the study period. This study illustrates the following: leading major diagnoses included malignant neoplasm of the head and neck, ulcerated and non-ulcerated inflammatory spectrum of chronic venous insufficiency, whereas angina pectoris, type 2 diabetes and cardiac diseases were noted most frequently as secondary diagnoses. Patients with venous diseases had considerably more often cardiopulmonary minor diagnoses, whereas endocrine diagnoses peaked in the cohort of patients with psoriasis and psychiatric and muscululoskeletal disorders in patients with bullous dieseases. Moh’s surgery, dressings and multimodal dermatological treatments were the most often encoded procedures.

**Supplementary Information:**

The online version contains supplementary material available at 10.1007/s00403-021-02244-9.

## Introduction

In Western Europe, the increase in life expectancy due to an increasingly higher health status and continuously improving medical care results in a growing number of elderly people, a trend that can also be observed in the age distribution of hospitalized patients [1–3]. This trend will be aggravated due to relatively low birth ratio and further expectable medical developments combined with the ageing “baby boomer” generation, the demographic cohort born after World War II, in Germany and elsewhere [4].

Current geriatrics literature focuses mainly on neuropsychiatric, orthopaedic or internal diseases [5–10], but limited data exist regarding hospitalizations due to dermatologic conditions in elderly German patients.

From a health planning and management perspective, characterising the demands and requirements of geriatric patients (65 years of age and older) in relation to specific challenges accompanying hospitalization and treatment, is crucial. Our work illustrates the burden of diseases severe enough for inpatient treatment.

## Materials and methods

The study was conducted retrospectively from January 1st, 2009 to December 31st, 2017 at the Department of Dermatology, University of Freiburg, Germany. It included all hospitalized patients who were 65 years or older at the time of the admission. The study was approved by the University of Freiburg Ethics Committee Board and registered on German Clinical Trials Register (http://www.drks.de, DRKS-ID DRKS00016865).

Data were collected and grouped in major and comorbidities based on electronically documented international classification of diseases (ICD)-10 codes in cooperation with the Department of Medical Controlling. If patients were hospitalized more than once in the investigated time period, each admission was included separately.

Subgroup analysis of the main diagnoses were done by clustering primarily surgical patients (nonmelanoma skin cancer (NMSC) and Merkel cell carcinoma (MCC) (ICD-10 codes C44, D04), and melanoma (C43, D03)), as well as non-surgical patients (psoriasis (L40), mature T/NK-cell lymphoma (C84), (autoimmune) bullous dermatoses (L10–L14), lupus erythematosus (L93), erysipelas (A46), zoster (B02), dermatitis and eczema (L20–L30), and venous disease with or without ulceration (I83)), and others.

Performed procedures were grouped according to OPS (‘Operationen- und Prozedurenschlüssel’)-301 categories (German modification of the International Classification of Procedures in Medicine).

Descriptive statistics (Wilcoxon–Mann–Whitney test [11] and Chi–square test [12]) were used to assess the data, and *t* test as well as anova tests followed by a Tukey multiple comparison of means test was performed for inductive statistics. Detailed calculations of significance can be found in Online Resource 2. Figures were created using R (v.4.0.2) and CorelDraw (v.21).

## Results

### Patient numbers

In the exemplary year 2016, 58,947 patients were hospitalized in the Medical Center—University of Freiburg, of which 23,986 patients were aged 65 years or older (40.7%). The Dermatological Department ranked fourth out of 26 of all inpatient departments regarding the percentage of geriatric patients (1192 patients or 53%), whereas the departments anaesthesiology (66%), ophthalmology (63%), and palliative medicine (54%) had the eldest patient collective.

Between 2009 and 2017, 10,009 individual geriatric hospitalizations were confirmed, of which 5346 patients presented once, while 1148 patients were hospitalized twice or more than twice (603 patients) during this timeframe.

**Fig. 1:**
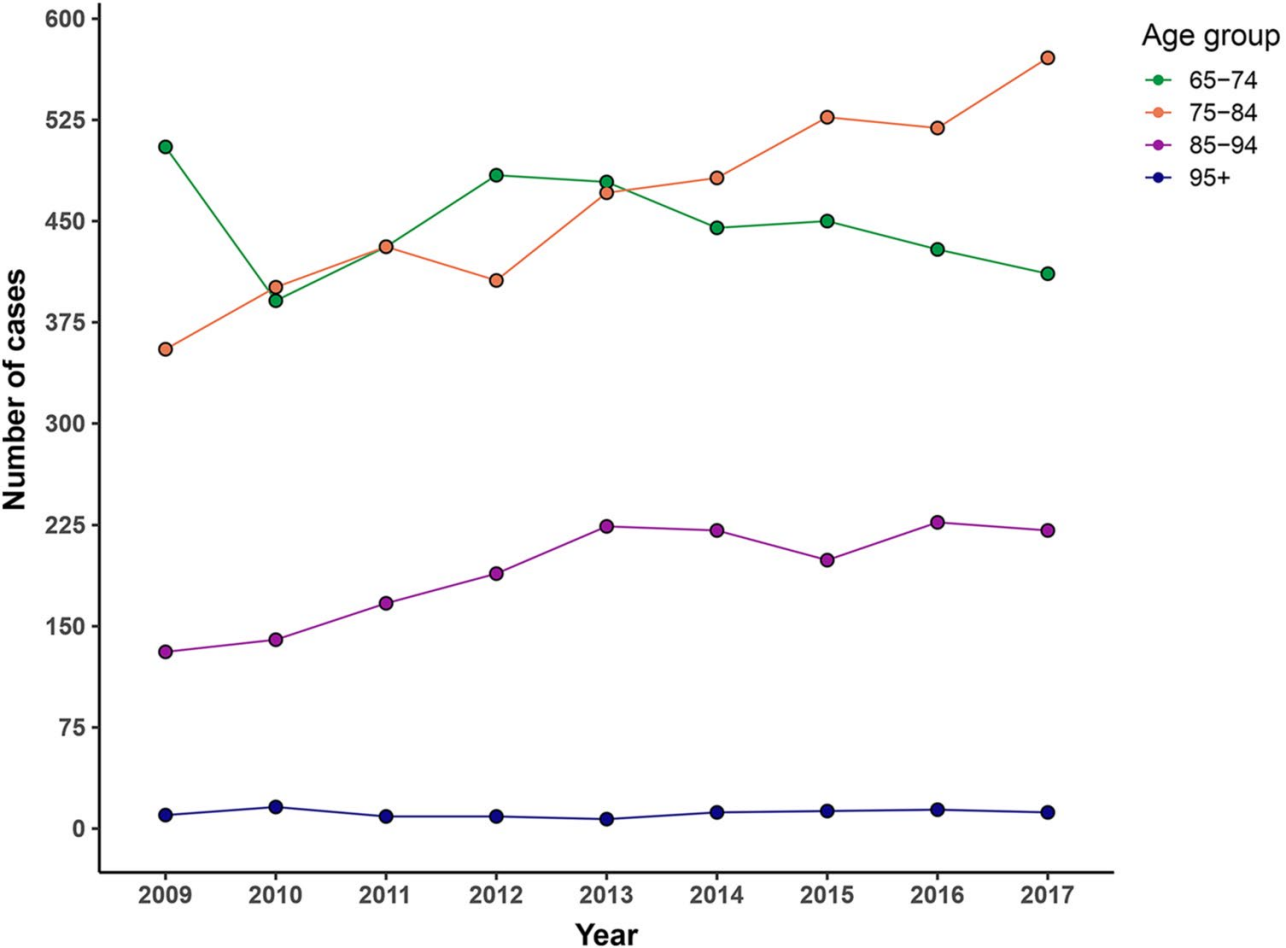
Number of individual hospitalizations of patients aged equal to and over 65 years between 2009 and 2017 Subgrouping according to age. Total *n* = 10,009

The overall number of geriatric hospitalizations rose from 1001 cases in 2009 to 1214 cases in 2017 (equivalent to a rise from 47.2 to 50.8% of all hospitalized cases, *p* < 0.01). Considering different age subgroups, the number of inpatients between 75 and 84 years as well as 85 and 94 years increased significantly in an even greater extent (17.2–23.9% and 6.3–9.2% of all in the same year hospitalized patients, *p* < 0.01) (Fig. 1).

### Gender

There was a slight predominance of male patients (51%), and males represent the majority in the age cohort 65- to 85-year-olds (54% of patients), whereas female patients make up for more than 61% of all patients aged older than 85 years.

### Main diagnoses

The most frequently encoded individual diagnoses identified were “other and unspecified malignant neoplasm of skin” (C44), “malignant melanoma of skin” (C43) and “varicose veins of lower extremities” (I83) (see Online Resource 1: Table 1A and B).

**Fig. 2:**
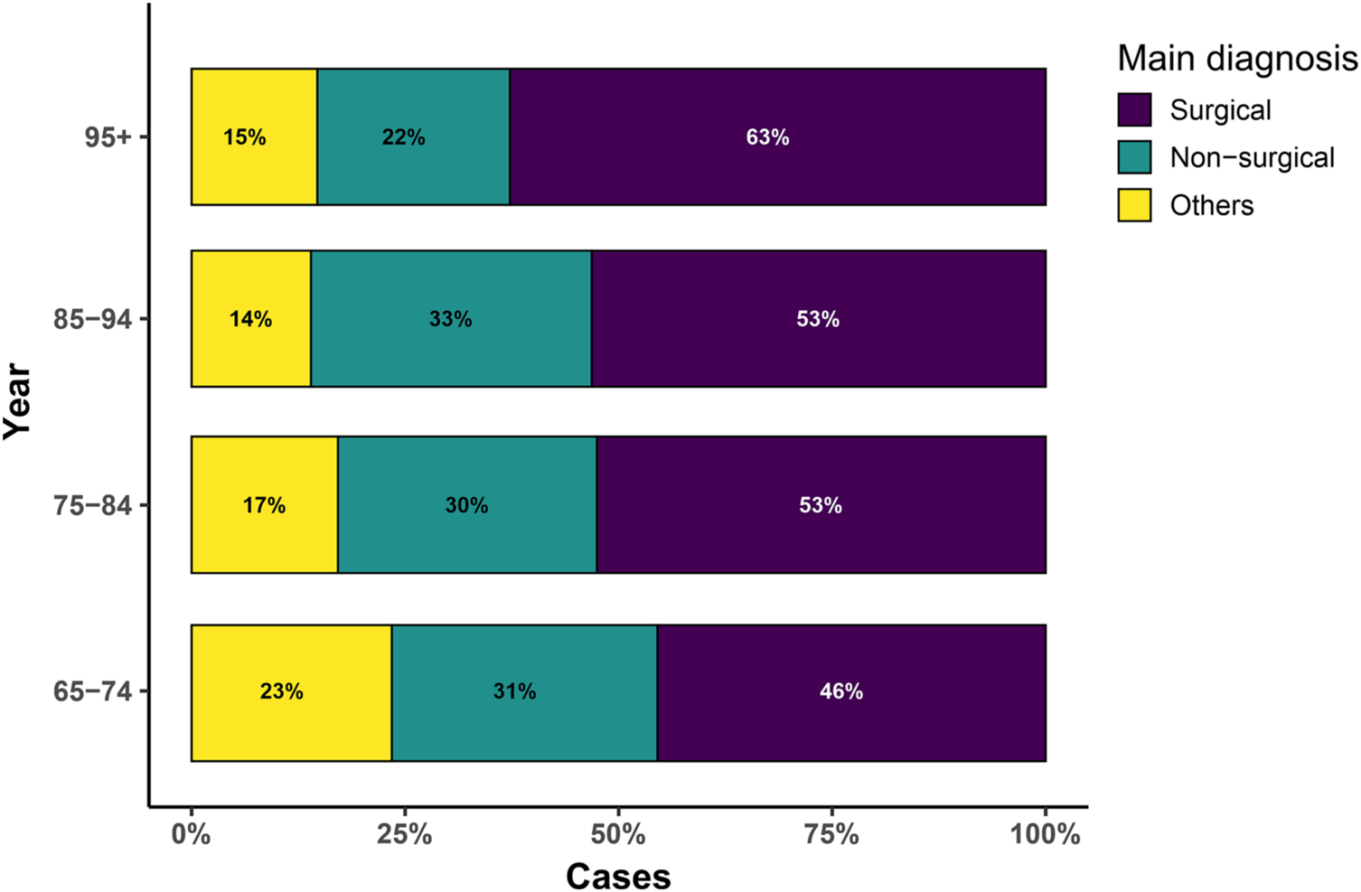
Subgroup analysis clustering primarily patients with operative diagnoses (NMSC/MCC/melanoma), non-operative diagnoses (psoriasis/matureT/NK-cell lymphoma/ (autoimmune) bullous dermatoses with lupus erythematosus/erysipelas/zoster/dermatitis/eczema/venous disease with/without ulceration), and all other remaining diagnoses of all individual hospitalizations of patients aged equal to and over 65 years between 2009 and 2017. Subgrouping according to age. Total *n* = 10,009. *NMSC* nonmelanoma skin cancer, *MCC* Merkel cell carcinoma

A subgroup analysis clustering indications in “surgical”, “non-surgical” and “other diagnoses” revealed a steady distribution over the study period (Fig. 2), but with a clear trend that older patients were hospitalized more frequently due to surgical diagnoses. Surgical patients make up for half of all geriatric cases, while non-surgical and other patients comprise about 30% and 20% of the cases, respectively.

Differences in the encoded main diagnoses in the year 2009 compared to 2017 showed the following: We had more hospitalizations for “atherosclerosis of arteries of extremities” associated ulcerations (I70.2), “atopic dermatitis” (L20) or “secondary and unspecified malignant neoplasm of lymph nodes” (C77) in 2009 (1.6% (2009) vs. 0.41% (2017), 3% vs. 0.9% (both *p* < 0.05); 2.6% vs. 1.2% (*p* = 0.086)), whereas diagnoses like “varicose veins of lower extremities” (I83), “non-pressure chronic ulcer of lower limb, not elsewhere classified” (L97) or “transepidermal elimination disorders” like reactive perforating collagenosis (I87) were far more often encoded in 2017 (3.2% vs. 6.7%; 1.7% vs. 3.7%; 0.1% vs. 1.7%, all *p* < 0.05, respectively).

### Age- and gender-specific distribution of main diagnoses

**Fig. 3:**
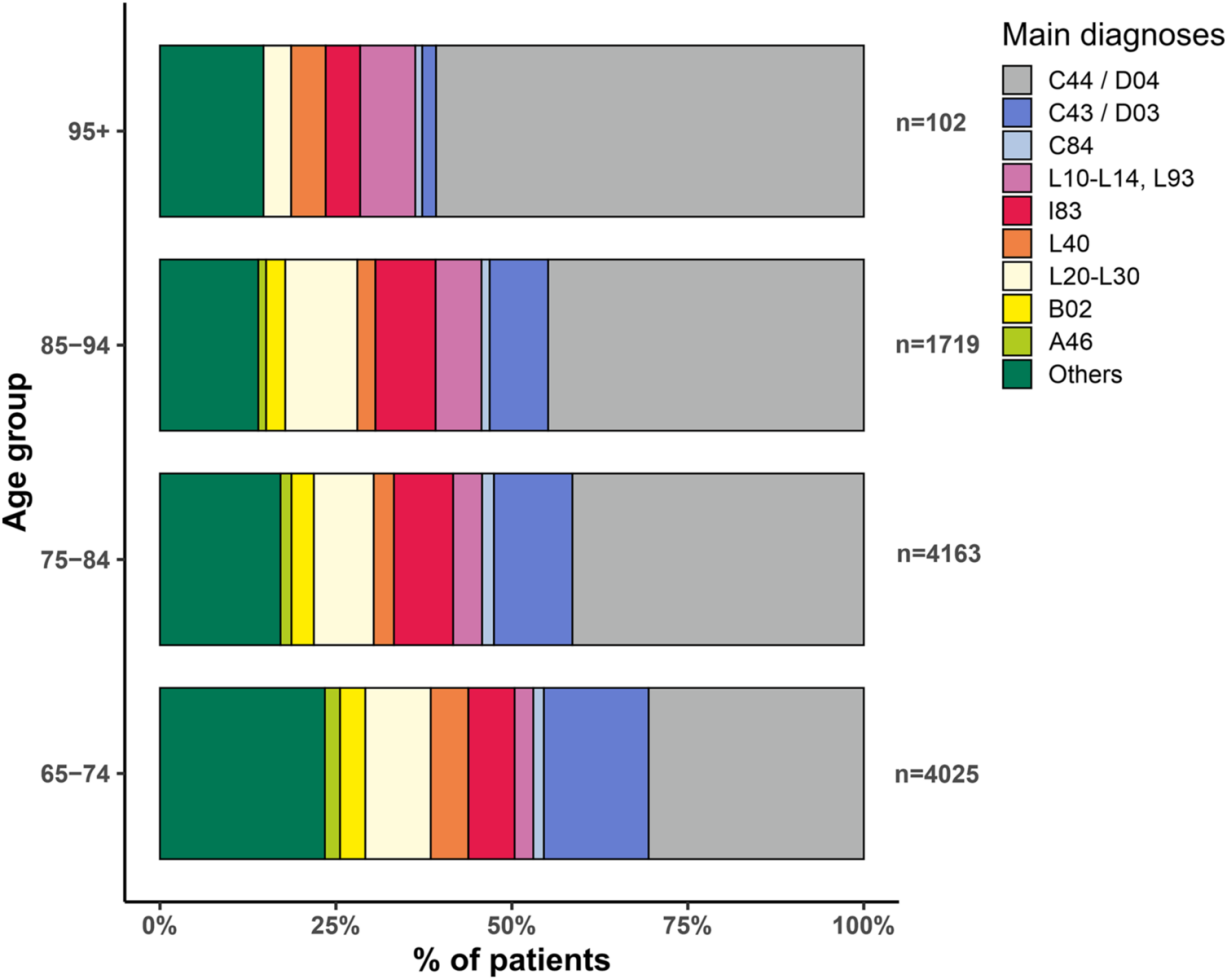
Subgroup analysis of specific diagnoses performed for different age cohorts between 2009 and 2017. Total *n* = 10,009. ICD-10 codes: nonmelanoma skin cancer/Merkel cell carcinoma (C44/D04), melanoma (C43/D03), mature T/NK-cell lymphoma (C84); (autoimmune) bullous dermatoses (L10-L14) including lupus erythematosus (L93), venous disease with/without ulceration (I83), psoriasis (L40), dermatitis/eczema (L20-L30), zoster (B02), erysipelas (A46)

Subgroup analysis showed that the numbers of patients treated for NMSC and MCC or pemphigoid rise with age (31% vs. 60.1% and 1.7% vs. 6.9% in patients aged 65 to 74 years vs. over 95 years, *p* < 0.01), whereas older patients were less often treated for malignant melanoma (11.9% vs. 2%, *p* < 0.05), or psoriasis (5.3% vs. no case, *p* < 0.01) (Fig. 3, Table 1).

**Table 1:** Frequency of the ten most commonly encoded main diagnoses of all individual hospitalizations of patients aged equal to and over 65 years between 2009 and 2017

65–74 years			75–84 years			85–94 years			≥ 95 years		
Main diagnosis	*n*	%	Main diagnosis	*n*	%	Main diagnosis	*n*	%	Main diagnosis	*n*	%
Malignant neoplasm of face^a,b^ (C44.3)	796	19.8	Malignant neoplasm of face^a,b^ (C44.3)	1074	25.8	Malignant neoplasm of face^a,b^ (C44.3)	492	28.6	Malignant neoplasm of face^a,b^ (C44.3)	30	29.4
Psoriasis vulgaris (L40.0)	203	5.0	Varicose veins of lower extremities with both ulcer and inflammation (I83.2)	310	7.4	Varicose veins of lower extremities with both ulcer and inflammation (I83.2)	129	7.5	Malignant neoplasm of scalp and neck^a^ (C44.4)	18	17.6
Varicose veins of lower extremities with both ulcer and inflammation (I83.2)	199	4.9	Malignant neoplasm of scalp and neck^a^ (C44.4)	261	6.3	Malignant neoplasm of scalp and neck^a^ (C44.4)	119	6.9	Malignant neoplasm of ear and external auricular canal^a^ (C44.2)	7	6.9
Malignant melanoma of lower limb, including hip (C43.7)	131	3.3	Malignant neoplasm of ear and external auricular canal^a^ (C44.2)	165	4.0	Bullous pemphigoid (L12.0)	100	5.8	Bullous pemphigoid (L12.0)	7	6.9
Malignant neoplasm of scalp and neck^a^ (C44.4)	130	3.2	Other specified dermatitis (L30.8)	142	3.4	Other specified dermatitis (L30.8)	86	5.0	Varicose veins of lower extremities with both ulcer and inflammation (I83.2)	4	3.9
Malignant melanoma of trunk (C43.5)	129	3.2	Bullous pemphigoid (L12.0)	131	3.1	Malignant neoplasm of lower limb, including hip^a^ (C44.7)	47	2.7	Zoster with other nervous system involvement (B02.2)	3	2.9
Other atopic dermatitis (L20.8)	106	2.6	Non-pressure chronic ulcer of lower limb, not elsewhere classified (L97)	112	2.7	Non-pressure chronic ulcer of lower limb, not elsewhere classified (L97)	46	2.7	Malignant neoplasm of external lower lip (C00.1)	3	2.9
Melanoma in situ of face^b^ (D03.3)	100	2.5	Psoriasis vulgaris (L40.0)	104	2.5	Malignant neoplasm of ear and external auricular canal^a^ (C44.2)	43	2.5	Other specified dermatitis (L30.8)	3	2.9
Other specified dermatitis (L30.8)	97	2.4	Malignant melanoma of face^b^ (C43.3)	89	2.1	Psoriasis vulgaris (L40.0)	37	2.2	Actinic keratosis (L57.0)	3	2.9
Malignant melanoma of upper limb, including shoulder (C43.6)	96	2.4	Malignant melanoma of lower limb, including hip (C43.7)	86	2.1	Malignant melanoma of face^b^ (C43.3)	33	1.9	Malignant neoplasm of trunk^a^ (C44.5)	2	2.0

Subgrouping according to age

^a^unspecified

^b^not elsewhere classified

^c^without ulcer or inflammation

Regarding gender, our data reveal more males hospitalized for melanoma (C43, D03) and NMSC (C44, D04) than females (53.7% vs. 44.5% of all registered male or female cases). Cutaneous malignancies localized on the skin of scalp and neck (C43.4, C44.4, D03.4) and the ear/external auricular canal (C43.2, C44.2, D03.2) were significantly more prominent in men than in women (scalp and neck: 9.4% vs. 2.5% of all registered male or female cases; ear/external auricular canal: 5.4% vs. 0.96%, respectively). Vice versa, there is a slight trend towards a more frequent occurrence of malignancies on the skin of the face (C43.4, C44.4, D03.4) in women than in men (30.4% vs. 25.7%, all *p* < 0.001).

Overall, some diagnoses are unevenly distributed: “Sézary syndrome” (C84.1) and “rhinophyma” (L71.1) occur in 1.49% and 0.42% of all male cases and are almost never registered in women (0.33% and 0.04%, respectively, *p* < 0.01). On the contrary, “pemphigoid” (L12), “bullous erythema multiforme” (L51.1), “pyoderma gangraenosum” (L88), and “discoid lupus erythematosus” (L93.0) are seen more often in women than in men (3.8% vs. 2.4%; 0.18% vs. 0.02% (both *p* < 0.01); 0.57% vs. 0.25%; 0.16% vs. 0.02% (both *p* < 0.05), respectively).

### Comorbidities

The 10,009 hospitalisation cases were encoded with 51,793 comorbidities. The most commonly encoded diagnoses were “angina pectoris” (I10, 6294 cases, 63% of all hospitalisation cases), “type 2 diabetes mellitus” (E11, 2059 cases, 21%), “skin changes due to chronic exposure to nonionizing radiation” (e.g. actinic keratosis, L57, 1757 cases. 18%) and “atrial fibrillation and flutter” (I48, 1619 cases, 16%) (see Online Resource 1: Table 2).

**Fig. 4:**
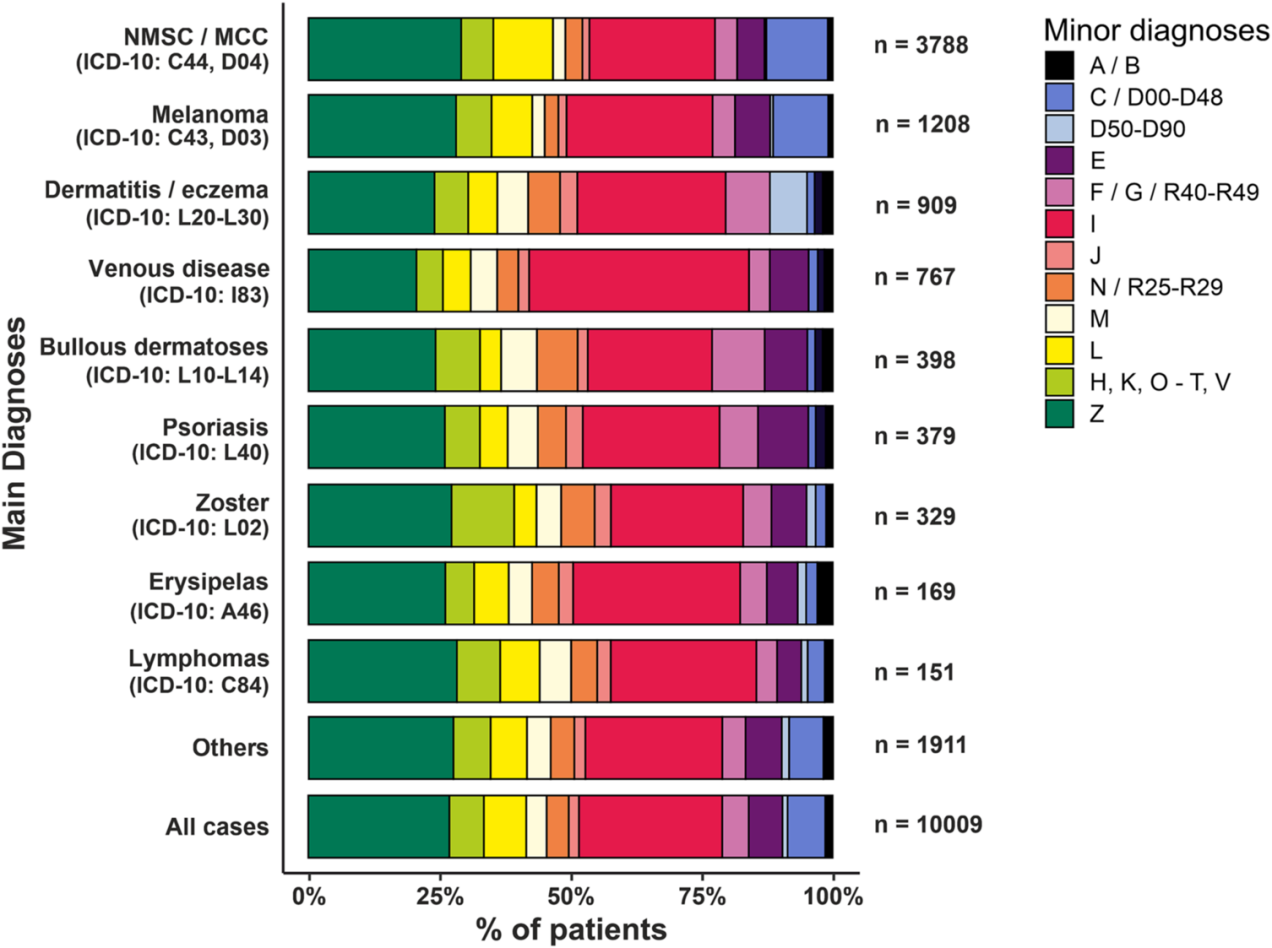
Enlistment of minor diagnoses grouped according to ICD-10 categories for individual main diagnoses of all individual hospitalizations of patients aged equal to and over 65 years between 2009 and 2017. ICD-10 of minor diagnoses: infectious /parasitic diseases (A/B), neoplasms (C/D00-D48), diseases of blood/immune system (D50-D90), endocrine/nutritional/metabolic diseases (E), psychiatric diseases/diseases of nervous system (ICD-10 F/G/R40-R49), diseases of circulatory/pulmonary system (I and J); diseases of skin (L), diseases of musculoskelettal system/connective tissue (M); diseases of genitourinary system (N/R25-R29), factors influencing health status/contact with health services (Z), other diagnoses (H, K, O, P, Q, R, S, T, V). *NMSC* nonmelanoma skin cancer, *MCC* Merkel cell carcinoma

Correlating the different minor diagnoses with groups of the main diagnoses, we found that patients with venous diseases have a higher frequency of circulatory and respiratory illnesses (44%) when compared to all diagnoses combined (27.5%), whereas patients with bullous disorders suffer more often from musculoskelettal (6.8%) as well as psychiatric diseases (10%), and patients diagnosed with psoriasis were diagnosed with more endocrine disorders (9.6%) than other diagnoses. Also, patients hospitalized due to NMSC/MCC or melanoma more often reported a comorbidity related to other neoplasms (11.4%) than patients with other major diagnoses (5.9%). (Fig. 4, Online Resource 1: Table 3).

Classically, ageing as “senility” is subsumed in the ICD-10 category R54. However, only 38 cases were registered with this minor diagnosis over the vast study period. Other diagnoses associated with older age like “abnormalities of gait and mobility” (R26; 600 cases), “tendency to fall” (R29.6; 500 cases), “(pre)senile dementia” (F03, 364 cases), “Alzheimer disease” (G30; 48 cases) or “presbycusis” (H91.1; 138 cases) were registered quite frequently (Table 2). Of notice, “unspecified urinary incontinence” (R32) and “faecal incontinence” (R15) were listed more frequently in women than in men and rise with increasing age (R32: 9.1% vs. 4.1% or all female or male cases; R15: 3.5% vs. 1.6%, *p* < 0.001) (Table 2).

**Table 2:** Registered frequencies of individual minor diagnoses associated with ageing of all individual hospitalizations of patients aged equal to and over 65 years between 2009 and 2017

Minor diagnoses	Total cases	Age cohorts in years															
		65–74				75–84				85–84				≥ 95			
		Male		Female		Male		Female		Male		Female		Male		Female	
		*n*	%	*n*	%	*n*	%	*n*	%	*n*	%	*n*	%	*n*	%	*n*	%
Urinary incontinence^a^ (R32)	658	36	1.6	67	3.8	98	4.5	156	7.9	65	9.8	212	20.0	11	25	13	22.4
Abnormalities of gait and mobility (R26)	600	47	2.1	70	3.9	90	4.1	169	8.5	47	7.0	157	14.8	6	13.6	14	24.1
Repeated falls (R29.6)	500	40	1.8	54	3.0	70	3.2	151	7.6	36	5.5	135	12.8	4	9.1	10	17.2
Dementia^a^ (F03)	364	20	0.9	15	0.8	67	3.1	80	4.0	52	7.9	117	11.1	3	6.8	10	17.2
Fecal incontinence (R15)	255	17	0.8	24	1.3	42	1.9	64	3.2	20	3.0	80	7.6	3	6.8	5	8.6
Presbycusis (H91.1)	138	14	0.6	8	0.5	24	1.1	20	1.0	26	3.9	41	3.9			5	8.6
Alzheimer's disease (G30)	48	4	0.2	3	0.2	10	0.5	5	0.3	4	0.6	20	1.9	1	2.3	1	1.7
Age-related physical debility (R54)	38	2	0.1	2	0.1	3	0.1	5	0.3	7	1.1	14	1.3	2	4.6	3	5.2

Subgrouping according to gender

^a^unspecified

### Age- and gender-specific distribution of comorbidities

With increasing age, urinary incontinence (R32) and dementia (F03) became far more frequent with older age (R32: 2.6% vs. 23.5% (65 to 74 years vs. aged 95 years or older); F03: < 1% vs. 12.7%). On the contrary, patients between 65 and 84 years regularly suffer from “obesity” (E66, about 10% of all cases), while only 5.2% of patients aged 85 years or older suffered from this complaint (Table 3).

**Table 3:** Frequency of the ten most commonly encoded minor diagnoses of all individual hospitalizations of patients aged equal to and over 65 years between 2009 and 2017

65–74 years			75–84 years			85–94 years			≥ 95 years		
Minor diagnosis	*n*	%	Minor diagnosis	*n*	%	Minor diagnosis	*n*	%	Minor diagnosis	*n*	%
Benign essential hypertension (I10.0)	2050	50.9	Benign essential hypertension (I10.0)	2663	64.0	Benign essential hypertension (I10.0)	1200	69.8	Benign essential hypertension (I10.0)	60	58.8
Diabetes type 2 without complications (E11.9)	666	16.5	Diabetes type 2 without complications (E11.9)	948	22.8	Diabetes type 2 without complications (E11.9)	282	16.4	Urinary incontinence^a^ (R32)	24	23.5
CPVI (I87.2)	367	9.1	Atherosclerotic heart disease (I25.1)	557	13.4	Urinary incontinence^a^ (R32)	277	16.1	Diabetes type 2 without complications (E11.9)	15	14.7
Atherosclerotic heart disease (I25.1)	316	7.9	CPVI (I87.2)	469	11.3	Atherosclerotic heart disease (I25.1)	262	15.2	Atrial fibrillation and atrial flutter^a^ (I48.9)	15	14.7
Other obesity (E66.8)	212	5.3	Atrial fibrillation and atrial flutter^a^ (I48.9)	372	8.9	Atrial fibrillation and atrial flutter^a^ (I48.9)	206	12.0	Atherosclerotic heart disease (I25.1)	14	13.7
Atrial fibrillation and atrial flutter^a^ (I48.9)	201	5.0	Persistent atrial fibrillation (I48.1)	316	7.6	CPVI (I87.2)	201	11.7	Tendency to fall^b^ (R29.6)	14	13.7
Varicose veins of lower extremities^c^ (I83.9)	198	4.9	Urinary incontinence^a^ (R32)	254	6.1	Tendency to fall^b^ (R29.6)	171	9.9	Dementia^a^ (F03)	13	12.7
COPD^a^ (J44.9)	164	4.1	Tendency to fall^b^ (R29.6)	221	5.3	Dementia^a^ (F03)	169	9.8	CPVI (I87.2)	10	9.8
Persistent atrial fibrillation (I48.1)	157	3.9	Varicose veins of lower extremities^c^(I83.9)	216	5.2	Persistent atrial fibrillation (I48.1)	135	7.9	Difficulty in walking^b^ (R26.2)	10	9.8
Obesity due to excess calories (E66.0)	155	3.9	Other obesity (E66.8)	205	4.9	Chronic kidney disease^a^ (N18.9)	117	6.8	Immobility (R26.3)	10	9.8

*COPD* chronic obstructive pulmonary disease, *CPVI* chronic peripheral venous insufficiency

Subgrouping according to age

^a^unspecified

^b^not elsewhere classified

^c^without ulcer or inflammation

A gender-specific comparison reveals that male patients have far more often a “personal history of malignant neoplasm” (Z85; 39.8% vs. 27% of all male or female cases) or “chronic ischemic heart disease” (I25; 21.6% vs. 10.9%, both *p* < 0.001)). Also, the numbers for “mental and behavioural disorders due to use of alcohol” (F11) or “tobacco” (F17) or “sleep disorders” including sleep apnoea (G47) are doubled in the male patient clientele.

Vice versa, women are affected more prominently from diagnoses like “seropositive and other rheumatoid arthritis” (M05 and M06, 2.5% vs. 0.49%) or “other noninfective disorders of lymphatic vessels and lymph nodes” (I89; 5.8% vs. 2.1%, both *p* < 0.001). Also, diagnoses like “volume depletion” (E86) or “other disorders of fluid, electrolyte and acid–base balance” (E87) occurred almost twice as much in women (*p* < 0.05 or *p* < 0.01, respectively). Psychiatric disorders such as “depressive episode” (F32) or “recurrent depressive disorder” (F33), “other anxiety disorders” (F41) and “reaction to severe stress, and adjustment disorders” (F43) were almost three times more frequent in women (*p* < 0.001).

### Procedures

**Fig. 5:**
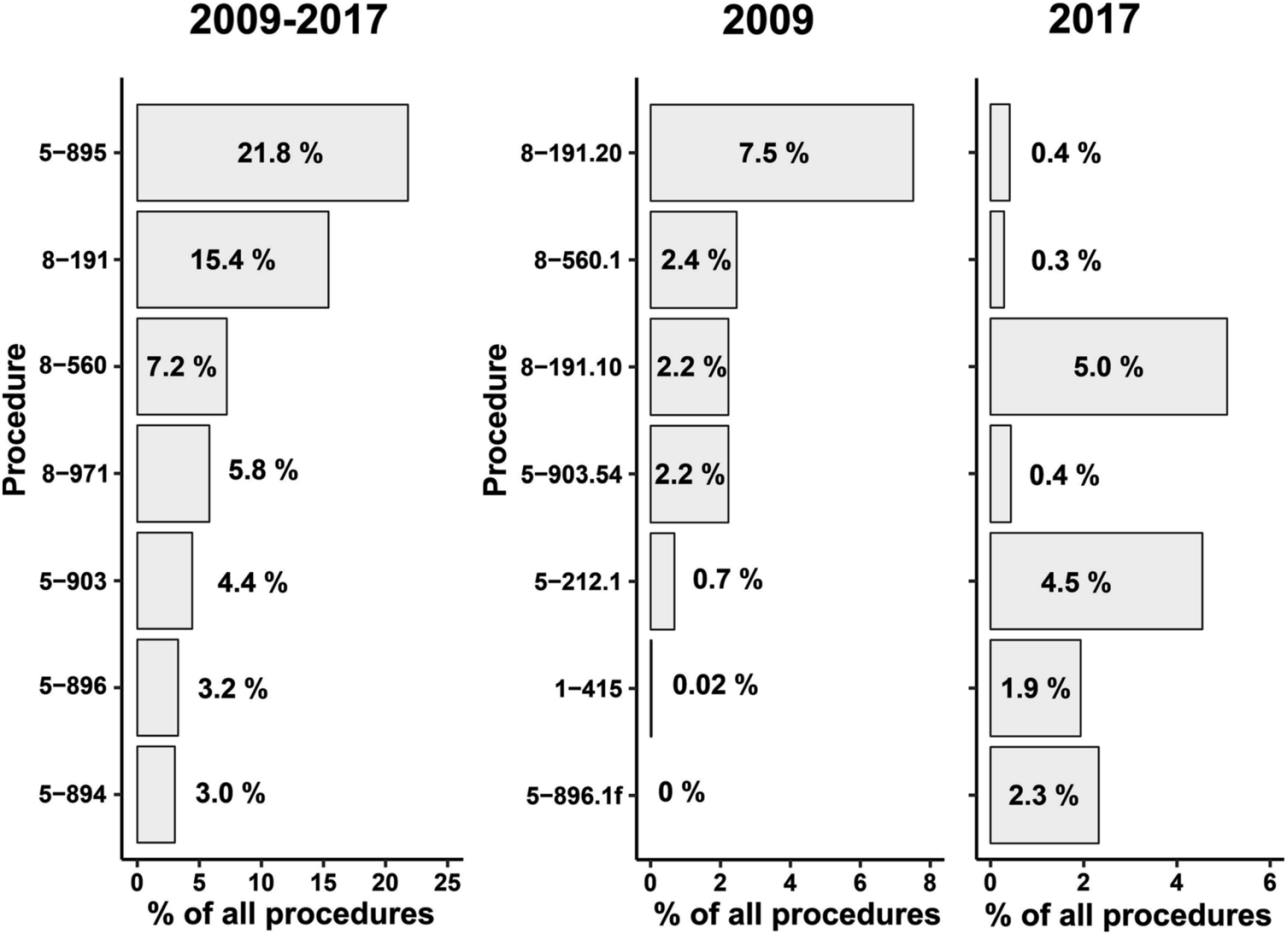
Numbers of most frequent encoded procedures 2009, 2017 and between 2009 and 2017. OPS-codes: “Moh’s surgery of diseased dermis and hypodermis”(5-895), “dressing of extensive and severe skin diseases” (8-191), “phototherapy” (8-560), “multimodal dermatological complex whole body treatment” (8-971), “regional skin flap” (5-903), “extensive debridement” (5-896), “paraffin gauze dressing with antiseptic ointments without debridement or bath” (8-191.20), “psoralen and ultraviolet A (PUVA) treatment)” (8-560.1), “paraffin gauze dressing with antiseptic solution without debridement or bath” (8-191.20) (8-191.10), “extensive local tissue expansion skin flap on the head” (5-903.54), “Moh’s surgery on the nasal skin” (5-212.1), “biopsy on the facial skin and the scalp” (1-415), “extensive debridement on the lower leg” (5-896.1f)

Overall, 34,876 procedures were registered. The main recoded procedures included “Moh’s surgery of diseased dermis and hypodermis” (OPS-2009: 5-895, 7604 cases, 21.8% of all events), “dressing of extensive and severe skin diseases” (8-191, 5,371 cases, 15.4%), “phototherapy” (8-560, 2514 cases, 7.2%), “multimodal dermatological complex whole body treatment” (8-971, 2024 cases, 5.8%), and “regional skin flap” (5-903, 1542 cases, 4.4%) (Fig. 5).

Between 2009 and 2017, there were only few differences in the most often encoded procedures. In 2009, more patients were encoded with procedures like “paraffin gauze dressing with antiseptic ointments without debridement or bath” (8-191.20; 7.5% in 2009 vs. 0.4% in 2017), “psoralen and ultraviolet A (PUVA) treatment” (8-560.1; 2.5% vs. 0.3%), and “extensive local tissue expansion skin flap on the head” (5-903.54, 2.2% vs. 0.4%). On the contrary, “paraffin gauze dressing without debridement or bath” (8-191.10; 5.1% in 2017 vs. 2.2% in 2009), “extensive debridement on the lower leg” (5-896.1f; 2.3% vs. 0%), “biopsy on the facial skin and the scalp” (1-415; 1.9% vs. 0.03%), and “Moh’s surgery on the nasal skin” (5-212.1; 4.5% vs. 0.7%) listed more frequently in 2017 (*p* < 0.001).

## Discussion

In Western Europe, the number of old (≥ 65 years) and very old (≥ 90 years) patients is expected to be rising, as the “baby boomer generation” (born in Germany between 1955 and 1969), is reaching the geriatric age and their needs will become a more important socioeconomically topic throughout all medical disciplines (Supplementary Material 1: figure 1). Whereas 21% of the German population in 2016 was 65 years or older, about 53% of the registered hospitalized patient cases belonged to this age cohort. In the next 15 years, the proportion of elderly is expected to increase to around 28% and the hypothetic rising number of geriatric inpatients requires more complex health care coordination.

A Germany study from Makrantonaki et al. exemplarily investigated all dermatological findings in inpatients of a geriatric unit and thereby gives an all-encompassing picture of the skin diseases older people suffer from, independent of the severity or need for inpatient treatment [13]. Since Germany is one of a few countries in which dermatology is an inpatient medical discipline, this study aims to map the most prevalent age associated skin disorders.

The observation that the top three registered diagnoses in men are all NMSC of different localisations confirms their increased risk for cutaneous malignancies with an additional clear surplus of all inpatient cases between 65 and 84 years [14, 15]. The surplus may be attributed in part to differences in occupation, tanning behaviour and sun protection, and naturally exposed areas is reflected in this study [16–19]. Female patients were also hospitalized for venous problems (with ulcerations) or dermatitis. In the cohort of over 95 year-olds we found a shift towards diseases associated with a significant reduction in the quality of life if left untreated, like progressively enlarging NMSC or pemphigoid disease, associated with infection, pain and bleeding, while less patients are recorded for inflammatory diseases like atopic dermatitis (Fig. 3).

The discrepancy of a clear surplus of hospitalized women in the group over 85-year-old in the gender distribution of our inpatients is clearly mirrored in the demographic distribution in Germany. Besides less risky health behaviour, this gender gap can be led back to male soldiers lost in the second world war, and may equally adapt due to missing social cuts in the meantime[20].

Nevertheless, the registered procedures match the found main diagnoses: With NMSC and melanoma being the most common reasons for hospitalization, Moh’s surgery was the most frequently encoded procedure, respectively. Similarly, dressings essential after both surgical procedures and for ulcerations are often recorded, as are specific dermatological therapies needed for disorders like dermatitis or psoriasis. But, as mentioned above, the incomplete documentation of the performed procedures makes it impossible to validly describe occurring trends throughout the years. That said, the observed shift in the frequencies of the registered measurements might be partly due to revisions of guidelines for melanoma, squamous cell carcinoma, pemphigus and bullous pemphigoid, and atopic dermatitis in the last years [15, 21–24].

For example, the comparably low number of very old patients with a hospitalization diagnosis of melanoma may, amongst other reasons like the overall lower incidence than NMSC or a higher mortality in earlier life, be associated with a decision against excessive treatment options such as lymph node extirpation and thus a more frequent ambulatory therapy.

Analysing the registered comorbidities, we saw that most frequently encoded diagnoses coincide in both sexes. These included cardiovascular diseases, venous diseases, diabetes mellitus type 2 or obesity, and malignancies, which represent the major burdens resulting from an unhealthy lifestyle with an excess in caloric intake and little exercise. Gender-specifically, we found that men enlist far more often with a “personal history of malignant neoplasm”, an ongoing observation of which Nicholas provides an excellent overview of possible reasons [25, 26]. Other examples of minor diagnoses that are found in gender-distinct frequencies are “mental and behavioural disorders due to use of alcohol” or “tobacco” in men, and depression disorders or “seropositive and other rheumatoid arthritis” in women [27–31]. Newly emerging therapies like the nowadays increasing use of targeted therapies will surely change the inpatient clientele for both therapeutic success with less hospitalizations (psoriasis or melanoma) as well as more admissions for the management of their possible side effects.

Astonishingly, age-related comorbidities are underrepresented in comparison to clinical reality, which may be attributed to tight time schedules, agitating clinical environment, “geriatically” untrained physicians or less relevant financial reimbursement, although these factors also contribute to the length of stay, complications, medical transfers and consultative support by other disciplines (data not shown).

In general, all registered diagnoses as well as the encoded procedures strongly link to the experience, continuity and consistency of the person responsible for coding. Yet, a structural bias was evident to the effect that not all, but foremost those for financial reimbursement needed diagnoses were recorded. Procedures performed at the ward that are intended for good clinical practice, are time-consuming and costly, but did not have OPS coding, were not encoded at all. This includes small biopsies or caretaking of a postoperative bleeding, blood workup in various areas, urine or pregnancy testing, microbiological testing or histological examinations, laser therapy, ultrasound of lymph node or duplex ultrasound for venous diseases. While this is a monocentric study, differences between individual medical facilities surely exist.

## Conclusion

Benefitting from a steep improvement and innovations in the health care system and, in comparison to former generations, better nutrition and less physical work, this age group is expected to reach a high age in considerable fitness. Therefore, it will be crucial not only to adapt to the physical need by senior-friendly modifying buildings, but also to create a modernized structured comprehensive all-encompassing registration system that serves as a foundation for data registration, transfer and extraction of diagnoses and procedures mentioned in medical treatment. New balances between an adequate financial recompensation for the highly complex in- and out-patient treatment of the aging society and the lack of temptation for health care providers to “sicken” their patients. Education of the medical staff for age-related changes and development of disorders, which includes recognition and diagnosing as well as the attendance to the specific requirements. In our opinion, a sensibilisation and medical education of the general population, which medical indications can still be treated meaningfully in high age, is crucial.

## Supplementary Information

Below is the link to the electronic supplementary material.

Supplementary Material 1 (Online Resource 1): A document with three tables and one figure. Table 1A lists the most common main diagnoses for male and female geriatric patients hospitalized in the study period. Table 1B lists the 20 most common main diagnoses grouped by main ICD-10 codes of the same patients. Table 2 lists the 20 most common minor diagnoses grouped by main ICD-10 codes for the same patients. Table 3 lists the frequency of minor diagnoses for certain main diagnoses for all patients of the study. Figure 1 compares the number of individual hospitalizations of elderly patients to the age distribution of Germany in 2017 (PDF 391 KB)

Supplementary Material 2 (Online Resource 1): A document that lists the results of statistical tests used to test for significance of results reported in the main document. (PDF 103 KB)

## Data Availability

Anonymised data are available from the corresponding author upon reasonable request.
