# Supplementary material for: Skin diseases in hospitalized geriatrics: a 9-year analysis from a University Dermatology Center in Germany
Source: Arch Dermatol Res. 2021 Jun 2;314(5):427–37. doi: 10.1007/s00403-021-02244-9 (PMC9163006; doi:10.1007/s00403-021-02244-9)

Supplementary Material A

Table 1 A: Enlistment of the 20 most common main diagnoses for males and females of all individual hospitalizations of patients aged equal to and over 65 years between 2009 and 2017 registered at the Department for Dermatology and Venerology in Freiburg.
*Other and unspecified of skin; ** of other and unspecified parts

Male patients (n = 5,109)				Female Patients (n = 4,900)			
**Main diagnosis**	**ICD-10**	**n**	**% of all registered male hospitalization cases**	**Main diagnosis**	**ICD-10**	**n**	**% of all registered female hospitalizati on cases**
Malignant neoplasm of face*,**	C44.3	1,164	22.78	Malignant neoplasm of face*,**	C44.3	1,227	25.05
Malignant neoplasm of scalp and neck*	C44.4	416	8.14	Varicose veins of lower extremities with both ulcer and inflammation	I83.2	399	8.14
Malignant neoplasm of ear and external auricular canal*	C44.2	259	5.07	Other specified dermatitis	L30.8	183	3.74
Varicose veins of lower extremities with both ulcer and inflammation	I83.2	243	4.76	Bullous pemphigoid	L12.0	180	3.67
Malignant melanoma of trunk	C43.5	181	3.54	Psoriasis vulgaris	L40.0	178	3.63
Psoriasis vulgaris	L40.0	165	3.29	Malignant melanoma of face**	C43.3	146	2.98
Other specified dermatitis	L30.8	144	2.82	Diagnostic testing of skin and sensibilisation	Z01.5	142	2.89
Other atopic dermatitis	L20.8	133	2.60	Non-pressure chronic ulcer of lower limb, not elsewhere classified	L97	142	2.89
Bullous pemphigoid	L12.0	121	2.37	Malignant melanoma of	C43.7	140	2.86

				lower limb, including hip			
Non-pressure chronic ulcer of lower limb, not elsewhere classified	L97	111	2.17	Melanoma in situ of face**	D03.3	118	2.41
Malignant neoplasm of lower limb, including hip*	C44.7	103	2.02	Malignant neoplasm of scalp and neck*	C44.4	112	2.29
Malignant melanoma of upper limb, including shoulder	C43.6	102	1.99	Malignant melanoma of upper limb, including shoulder	C43.6	109	2.22
Malignant melanoma of lower limb, including hip	C43.7	101	1.98	Erysipelas	A46	96	1.96
Malignant neoplasm of upper limb, including shoulder*	C44.6	86	1.68	Malignant neoplasm of lower limb, including hip*	C44.7	94	1.92
Melanoma in situ of face**	D03.3	78	1.53	Zoster with other nervous system involvement	B02.2	92	1.88
Zoster with other nervous system involvement	B02.2	78	1.53	Other atopic dermatitis	L20.8	81	1.65
Sézary disease	C84.1	76	1.49	Atherosclerosis of native arteries of right leg with ulceration	I70.23	60	1.22
Malignant neoplasm of trunk*	C44.5	73	1.43	Encounter for desensitization to allergens	Z51.6	58	1.18
Erysipelas	A46	73	1.43	Other prurigo	L28.2	55	1.12
Malignant melanoma of face**	C43.3	71	1.39	Secondary malignant neoplasm*	C79.2	55	1.12

Table 2 B: Enlistment of the 20 most common main diagnoses subgrouped according to ICD-10 code for males and females of all individual hospitalizations of patients aged equal to and over 65 years between 2009 and 2017 registered at the Department for Dermatology and Venerology in Freiburg.
*Other and unspecified of skin; ** of other and unspecified parts

**Diagnoses for male patients**				**Diagnoses for female patients**			
	**ICD-10**	**n**	**% of all registered male hospitalization cases (n = 5,109)**		**ICD-10**	**n**	**% of all registered female hospitalization cases (n = 4,900)**
Other and unspecified malignant neoplasm of skin	C44	2123	41.55	Other and unspecified malignant neoplasm of skin	C44	1594	32.53
Malignant melanoma of skin	C43	527	10.32	Varicose veins of lower extremities	I83	460	9.9
Varicose veins of lower extremities	I83	307	6.01	Malignant melanoma of skin	C43	449	9.16
Other and unspecified dermatitis	L30	219	4.29	Other and unspecified dermatitis	L30	255	5.2
Psoriasis	L40	176	3.44	Psoriasis	L40	203	4.14
Zoster	B02	157	3.07	Pemphigoid	L12	186	3.8
Atopic dermatitis	L20	133	2.6	Zoster	B02	172	3.51
Pemphigoid	L12	124	2.43	Non-pressure chronic ulcer of lower limb, not elsewhere classified	L97	142	2.9
Non-pressure chronic ulcer of lower limb, not elsewhere classified	L97	111	2.17	Other specific examination of patients without disorders or stated diagnosis	Z01	142	2.9
Mature T/NK-cell lymphomas	C84	104	2.04	Melanoma in situ	D03	137	2.8
Melanoma in situ	D03	95	1.86	Erysipelas	A46	96	1.96
Secondary and unspecified malignant neoplasm of lymph nodes	C77	95	1.86	Atopic dermatitis	L20	82	1.67

Erysipelas	A46	73	1.43	Atherosclerosis	I70	72	1.47
Secondary malignant neoplasm of other and unspecified sites	C79	69	1.35	Lichen simplex chronicus and prurigo	L28	71	1.45
Malignant neoplasm of lip	C00	67	1.31	Encounter for other aftercare and medical care	Z51	58	1.18
Atherosclerosis	I70	63	1.23	Secondary and unspecified malignant neoplasm of lymph nod	C77	57	1.16
Other specific examination of patients without disorders or stated diagnosis	Z01	54	1.06	Secondary malignant neoplasm of other and unspecified sites	C79	55	1.12
Encounter for other aftercare and medical care	Z51	46	0.9	Malignant neoplasm of lip	C00	52	1.06
Rosacea	L71	42	0.82	Mature T/NK-cell lymphomas	C84	47	0.96
Complications of procedures, not elsewhere classified	T81	31	0.61	Carcinoma in situ of skin	D04	36	0.73

Table 2: Enlistment of the 20 most common minor diagnoses subgrouped according to ICD-10 code for males and females of all individual hospitalizations of patients aged equal to and over 65 years between 2009 and 2017 registered at the Department for Dermatology and Venerology in Freiburg. Diagnoses encoded with ICD-10 category Z were excluded in the enlisting.
*unspecified; **other
COPD: Chronic obstructive pulmonary disease

**Diagnoses of male patients**				**Diagnoses for female patients**			
	**ICD-10**	**n**	**% of all registered male cases (n = 5,109)**		**ICD-10**	**n**	**% of all registered female hospitalization cases (n = 4,900)**
Angina pectoris	I10	3156	61.77	Angina pectoris	I10	3,138	64.05
Skin changes due to chronic exposure to nonionizing radiation	L57	1172	22.94	Type 2 diabetes mellitus	E11	951	19.41
Type 2 diabetes mellitus	E11	1108	21.69	Atrial fibrillation and flutter	I48	731	14.92
Chronic ischemic heart disease	I25	1105	21.63	Disorders of veins**	I87	723	14.76
Malignant neoplasm of skin*,**	C44	1099	21.51	Skin changes due to chronic exposure to nonionizing radiation	L57	585	11.94
Atrial fibrillation and flutter	I48	888	17.38	Chronic ischemic heart disease	I25	534	10.90
Chronic kidney disease	N18	598	11.70	Malignant neoplasm of skin*,**	C44	506	10.33
Complications of procedures, not elsewhere classified	T81	579	11.33	Varicose veins of lower extremities	I83	476	9.72
Disorders of veins**	I87	535	10.47	Overweight and obesity	E66	469	9.57
Overweight and obesity	E66	420	8.22	Urinary incontinence*	R32	448	9.14
Varicose veins of lower extremities	I83	419	8.2	Abnormalities of gait and mobility	R26	410	8.37
Carcinoma in situ of skin	D04	414	8.10	Chronic kidney disease	N18	395	8.06
COPD**	J44	279	5.5	Complications of procedures,	T81	386	7.88

				not elsewhere classified			
Malignant melanoma of skin	C43	240	4.7	Pain*	R52	351	7.16
Atherosclerosis	I70	230	4.50	Symptoms and signs involving the nervous and musculoskeletal systems**	R29	350	7.14
Sleep disorders	G47	216	4.23	Noninfective disorders of lymphatic vessels and lymph nodes**	I89	285	5.82
Urinary incontinence*	R32	210	4.11	Carcinoma in situ of skin	D04	270	5.51
Pain*	R52	204	3.99	Heart failure	I50	268	5.47
Heart failure	I50	203	3.97	Dermatitis*,**	L30	231	4.72
Seborrheic keratosis	L82	192	3.76	Dementia*	F03	222	4.53

Table 3: Frequency of the minor diagnoses grouped according to ICD-10 categories for chosen main diagnoses for all individual hospitalizations of patients aged equal to and over 65 years between 2009 and 2017 at the Department for Dermatology and Venerology in Freiburg.

	**NMSC / MCC** (C44 and D04)		**Melanoma** (C43 and D03)		**Lymphoma** (C84)		**Bullous dermatoses** (L10-L14 and L93)	
**Minor Diagnoses**	**n**	**%**	**n**	**%**	**n**	**%**	**n**	**%**
**Infectious / parasitic diseases** (ICD-10: A and B)	183	1.0	41	0.9	9	1.5	50	1.9
**Neoplasms** (ICD-10: C and D00-D48))	2246	11.7	492	10.5	19	3.3	37	1.4
**Diseases of blood/immune system** (ICD-10: D50-D90)	67	0.3	27	0.6	7	1.2	38	1.5
**Endocrine/nutritional/metabolic** Diseases (ICD-10: E)	999	5.2	314	6.7	27	4.6	210	8.1
**Psychiatric diseases/diseases of** nervous system (ICD-10: F, G and R40-R49)	808	4.2	200	4.3	23	3.9	258	10.0
**Diseases of circulatory system** (ICD-10: I)	4587	23.9	1305	27.9	162	27.8	611	23.7
**Diseases of pulmonary system** (ICD-10: J)	256	1.3	73	1.6	15	2.6	50	1.9
**Diseases of skin** (ICD-10: L)	2178	11.4	364	7.8	44	7.5	104	4.0
**Diseases of musculoskelettal system / connective tissue** (ICD-10: M)	454	2.4	111	2.4	35	6.0	176	6.8
**Diseases of genitourinary system** (ICD-10: N and R25-R29)	624	3.3	122	2.6	29	5.0	200	7.8
**Factors influencing health status / contact with health services** (ICD-10: Z)	5575	29.1	1318	28.1	165	28.3	624	24.2
**Other diagnoses** (ICD-10: H, K, O, P, Q, R, S, T, V)	1174	6.1	316	6.7	48	8.2	218	8.5

	Venous disease (I83)		Psoriasis (L40)		Dermatitis / eczema (L20-L30)		Zoster (B02)		Erysipelas (A46)		Other main diagnoses	
**Minor Diagnoses**	**n**	**%**	**n**	**%**	**n**	**%**	**n**	**%**	**n**	**%**	**n**	**%**
**Infectious / parasitic diseases** (ICD-10: A and B)	89	1.6	25	1.4	85	1.9	17	1.3	29	2.9	195	1.8
**Neoplasms** (ICD-10: C and D00-D48))	69	1.2	35	1.9	70	1.6	26	2.0	21	2.1	697	6.5
**Diseases of blood/immune system**(ICD-10: D50-D90)	97	1.8	26	1.4	64	1.4	23	1.7	16	1.6	150	1.4
**Endocrine/nutritional/metabolic** Diseases (ICD-10: E)	9	7.4	177	9.6	316	7.1	88	6.7	58	5.9	734	6.9
**Psychiatric diseases/diseases of nervous system** (ICD-10: F, G and R40-R49)	219	4.0	135	7.3	376	8.4	71	5.4	50	5.1	472	4.4
**Diseases of circulatory system** (ICD-10: I and J)	2317	41.9	481	26.1	1263	28.3	332	25.2	314	31.8	2781	26.1
**Diseases of pulmonary system** (ICD-10: J)	114	2.1	59	3.2	147	3.3	41	3.1	27	2.7	225	2.1
**Diseases of skin** (ICD-10: L)	293	5.3	98	5.3	248	5.6	56	4.2	65	6.6	740	7.0
**Diseases of musculoskelettal system / connective tissue** (ICD-10: M)	279	5.1	106	5.8	262	5.9	62	4.7	44	4.5	482	4.5
**Diseases of genitourinary system** (ICD-10: N and R25-R29)	223	4.0	99	5.4	271	6.1	84	6.4	50	5.1	479	4.5
**Factors influencing health status / contact with health services** (ICD-10: Z)	1,137	20.6	479	26.0	1,071	24.0	359	27.2	257	26.0	2936	27.6
**Other diagnoses** (ICD-10: H, K, O, P, Q, R, S, T, V)	278	5,0	123	6.7	287	6.4	157	11.9	54	5.5	754	7.1

Figure 1: Absolute number of individual hospitalizations of patients aged equal to and over 65 years between 2009 and 2017 registered at the Department for Dermatology and Venerology in Freiburg. Subgrouping according to gender. Underlying age distribution of Germany in 2017 (33). Total n = 10,009

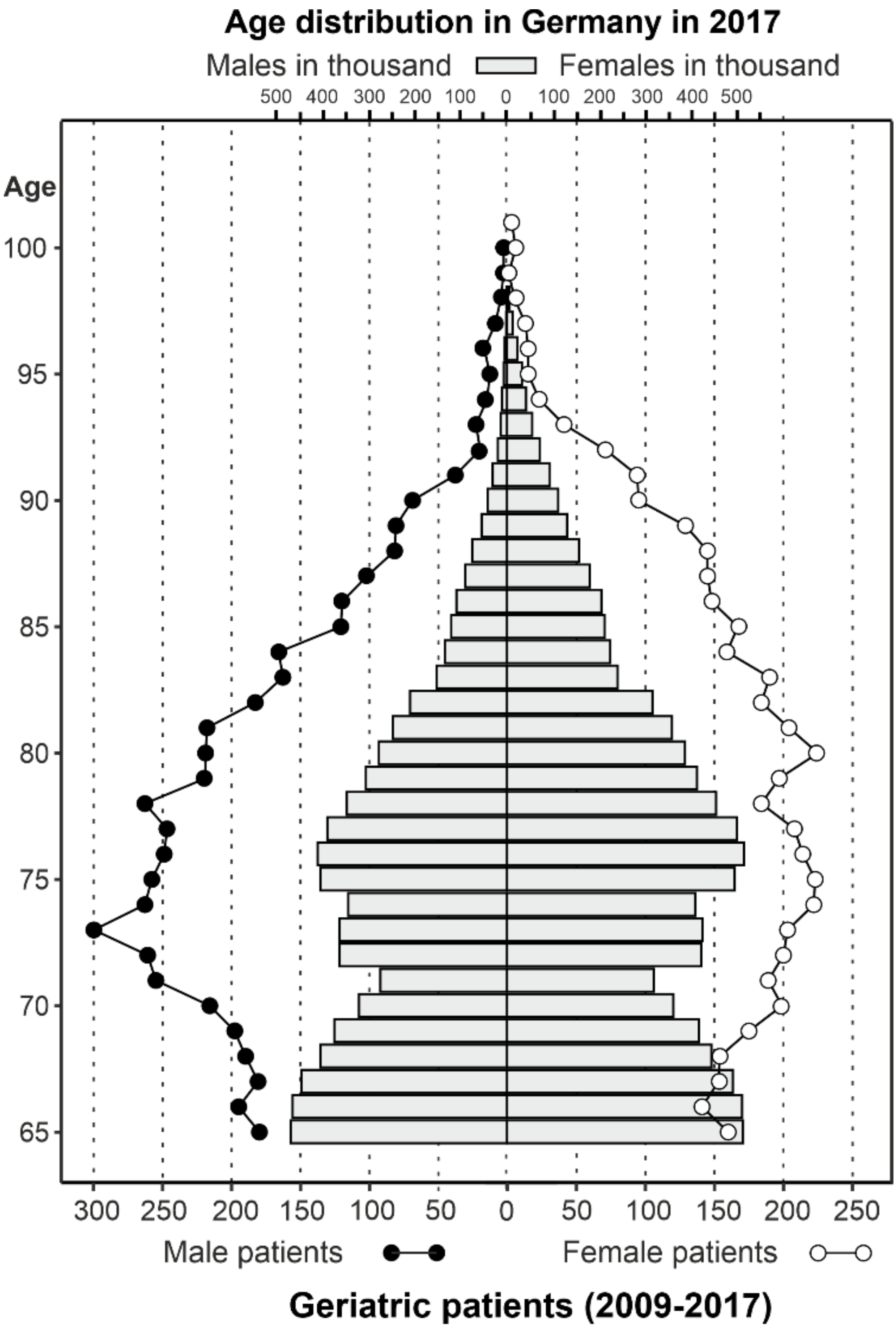

Supplement: Supplementary file 1 — Supplementary Material 1 (Online Resource 1): A document with three tables and one figure. Table 1A lists the most common main diagnoses for male and female geriatric patients hospitalized in the study period. Table 1B lists the 20 most common main diagnoses grouped by main ICD-10 codes of the same patients. Table 2 lists the 20 most common minor diagnoses grouped by main ICD-10 codes for the same patients. Table 3 lists the frequency of minor diagnoses for certain main diagnoses for all patients of the study. Figure 1 compares the number of individual hospitalizations of elderly patients to the age distribution of Germany in 2017 (PDF 391 KB) [file 403_2021_2244_MOESM1_ESM.pdf]
