# Supplementary material for: Skin diseases in hospitalized geriatrics: a 9-year analysis from a University Dermatology Center in Germany
Source: Arch Dermatol Res. 2021 Jun 2;314(5):427–37. doi: 10.1007/s00403-021-02244-9 (PMC9163006; doi:10.1007/s00403-021-02244-9)
Supplement: Supplementary file 2 — Supplementary Material 2 (Online Resource 1): A document that lists the results of statistical tests used to test for significance of results reported in the main document. (PDF 103 KB) [file 403_2021_2244_MOESM2_ESM.pdf]

Supplementary Material 2

In the following the results of statistical tests used to test for significance of results reported in the main manuscript text can be found. They are ordered according to position in the main text.

Patient numbers

Increase in geriatric patients from 2009 (1001 cases) to 2017 (1214 cases): $X^2(1, n = 2215) = 119.5, p < 0.01$

Increase of patients aged 75-84 years from 2009 (355 cases) to 2017 (571 cases): $X^2(1, n = 926) = 30.1, p < 0.01$

Increase of patients aged 85-94 years from 2009 (131 cases) to 2017 (221 cases): $X^2(1, n = 352) = 12.8, p < 0.01$

Main diagnoses

NMSC and MCC cases in the 65- to 74-year-old cohort (1,231 cases) vs the over-95-year-olds (62 cases):$X^2(1, n = 1293) = 42.2, p < 0.01$

Pemphigoid cases in the 65- to 74-year-old cohort vs the over-95-year-olds :$X^2(1, n = 75) = 14.9, p < 0.01$

Malignant melanoma in the 65- to 74-year-old cohort vs. the over-95-year-olds: $X^2(1, n = 215) = 5.7, p < 0.05$

Psoriasis in the 65- to 74-year-old cohort vs. the over-95-year-olds: $X^2(1, n = 601) = 13.3, p < 0.01$

Sézary syndrome" (C84.1) and "rhinophyma" (L71.1) in male patients vs female patients: $X^2(1, n = 92) = 37, p < 0.01$), $X^2(1, n = 42) = 36, p < 0.01$.

Pemphigoid (L12) in female vs male patients: $X^2(1, n = 310) = 15.6, p < 0.01$

Pyoderma gangraenosum (L88) in female vs male patients: $X^2(1, n = 41) = 6.2, p < 0.05$

Bullous erythema multiforme (L51.1) in female vs male patients: $X^2(1, n = 10) = 6.7, p < 0.01$

Discoid lupus erythematosus (L93.0) in female vs male patients: $X^2(1, n = 9) = 5.7, p < 0.05$)

Cutaneous malignancies localized on the skin of scalp and neck (ICD-10: C43.4, C44.4, D03.4) in male vs female patients: $X^2(1, n = 602) = 218, p < 0.001$

Cutaneous malignancies localized on the ear and external auricular canal (C43.2, C44.2, D03.2) in male vs female patients: $X^2(1, n = 325) = 164, p < 0.001$

Cases with atherosclerosis of arteries of extremities associated with ulcerations in 2009 vs 2017: $X^2(1, n = 21) = 5.76, p < 0.05$

Cases with atopic ulcerations in 2009 vs 2017: $X^2(1, n = 41) = 8.8, p < 0.05$

Cases with secondary and unspecified malignant neoplasm of lymph nodes in 2009 vs 2017: $X^2(1, n = 41) = 2.95, p = 0.086$),

Cases with varicose veins of lower extremities in 2009 vs 2017: $X^2(1, n = 113) = 21.25, p < 0.05$

Cases with non-pressure chronic ulcer of lower limb, not elsewhere classified in 2009 vs 2017: $X^2(1, n = 62) = 12.65, p < 0.05$

Cases with transepidermal elimination disorders like reactive perforating collagenosis in 2009 vs 2017: $X^2(1, n = 22) = 18.18, p < 0.05$)

Minor diagnoses

Personal history of malignant neoplasm (Z85) in male vs female cases: $X^2(1, n = 3357) = 148.06$, $p < 0.001$.

Chronic ischemic heart disease (I25) in male vs female cases: $X^2(1, n = 1639) = 198.93$, $p < 0.001$.

Seropositive rheumatoid arthritis (M05) and other rheumatoid arthritis (M06) in female vs male cases: $X^2(1, n = 144) = 64$, $p < 0.001$.

Other noninfective disorders of lymphatic vessels and lymph nodes (I89) in female vs male cases: $X^2(1, n = 390) = 83.08$, $p < 0.001$)

Volume depletion (E86) or other disorders of fluid, electrolyte and acid-base balance (E87) in female vs male cases: $X^2(1, n = 28) = 6.2$, $p < 0.05$, $X^2(1, n = 108) = 8.7$, $p < 0.01$.

Depressive episode (F32) in female vs male cases: $X^2(1, n = 183) = 62.6$, $p < 0.001$.

Recurrent depressive disorder (F33) in female vs male cases: $X^2(1, n = 227) = 52.3$, $p < 0.001$.

Other anxiety disorders (F41) in female vs male cases: $X^2(1, n = 31) = 13.3$, $p < 0.001$.

Reaction to severe stress, and adjustment disorders (F43) in female vs male cases: $X^2(1, n = 76) = 22.4$, $p < 0.001$.

Unspecified urinary incontinence (R32) in female vs male cases: $X^2(1, n = 658) = 86.08$, $p < 0.001$.

Faecal incontinence (R15) in female vs male cases: $X^2(1, n = 255) = 32.475$, $p < 0.001$.

Procedures

Paraffin gauze dressing with antiseptic ointments without debridement or bath (OPS-2019: 8-191.20) in 2009 vs 2017: $X^2(1, n = 304) = 272.6$, $p < 0.001$.

Psoralen and ultraviolet A (PUVA) treatment" (OPS-2019: 8-560.1) in 2009 vs 2017: $X^2(1, n = 106) = 71.2$, $p < 0.001$.

Extensive local tissue expansion skin flap on the head" (OPS-2019: 5-903.54) in 2009 vs 2017: $X^2(1, n = 103) = 49.8$, $p < 0.001$.

Paraffin gauze dressing without debridement or bath2 (OPS-2019: 8-191.10) in 2017 vs. 2009: $X^2(1, n = 295) = 45.2$, $p < 0.001$.

Extensive debridement on the lower leg (OPS-2019: 5-896.1f) in 2017 vs 2009: $X^2(1, n = 96) = 89.7$, $p < 0.001$.

Biopsy on the facial skin and the scalp (OPS -2019: 1-415) in 2017 vs. 2009: $X^2(1, n = 81) = 71.7$, $p < 0.001$.

Moh’s surgery on the nasal skin (OPS -2019: 5-212.1) in 2017 vs. 2009: $X^2(1, n = 214) = 113.3, p < 0.001$)).
